# Comparison of automated candidate gene prediction systems using genes implicated in type 2 diabetes by genome-wide association studies

**DOI:** 10.1186/1471-2105-10-S1-S69

**Published:** 2009-01-30

**Authors:** Erdahl T Teber, Jason Y Liu, Sara Ballouz, Diane Fatkin, Merridee A Wouters

**Affiliations:** 1 Victor Chang Cardiac Research Institute, 384 Victoria St, Darlinghurst, 2010, NSW, Australia; 2 School of Medical Sciences, University of New South Wales, Sydney, Australia

## Abstract

**Background:**

Automated candidate gene prediction systems allow geneticists to hone in on disease genes more rapidly by identifying the most probable candidate genes linked to the disease phenotypes under investigation. Here we assessed the ability of eight different candidate gene prediction systems to predict disease genes in intervals previously associated with type 2 diabetes by benchmarking their performance against genes implicated by recent genome-wide association studies.

**Results:**

Using a search space of 9556 genes, all but one of the systems pruned the genome in favour of genes associated with moderate to highly significant SNPs. Of the 11 genes associated with highly significant SNPs identified by the genome-wide association studies, eight were flagged as likely candidates by at least one of the prediction systems. A list of candidates produced by a previous consensus approach did not match any of the genes implicated by 706 moderate to highly significant SNPs flagged by the genome-wide association studies. We prioritized genes associated with medium significance SNPs.

**Conclusion:**

The study appraises the relative success of several candidate gene prediction systems against independent genetic data. Even when confronted with challengingly large intervals, the candidate gene prediction systems can successfully select likely disease genes. Furthermore, they can be used to filter statistically less-well-supported genetic data to select more likely candidates. We suggest consensus approaches fail because they penalize novel predictions made from independent underlying databases. To realize their full potential further work needs to be done on prioritization and annotation of genes.

## Background

The process of linking genes to disease phenotypes is rapidly gaining momentum since the first disease-causing gene was identified 25 years ago [1]. Alternative approaches adopted in the past to identify disease genes are the candidate gene approach, where likely suspects are prioritised and screened on a genome-wide basis; and linkage analysis where specific loci are determined systematically using family studies. The two approaches have been synthesized into a pipeline by completion of the Human Genome Project; and further enabled by the increased availability of high-throughput experimental data and the development of sophisticated bioinformatics tools. In addition there have been efforts in the bioinformatics community to systematize and automate candidate gene prediction. Automated prediction systems provide geneticists with a reduced list of genes estimated to have a high probability of involvement in the disease phenotype by sifting through hundreds to thousands of genes. Ultimately, these tools aim to give the researcher the best possible guidance in honing in on the gene culprits for further biological confirmation. Since their introduction in the early 2000s, the predictive powers of automated candidate gene prediction systems have improved, largely due to increases in biological systems knowledge and more effective algorithms.

**Figure 1:**
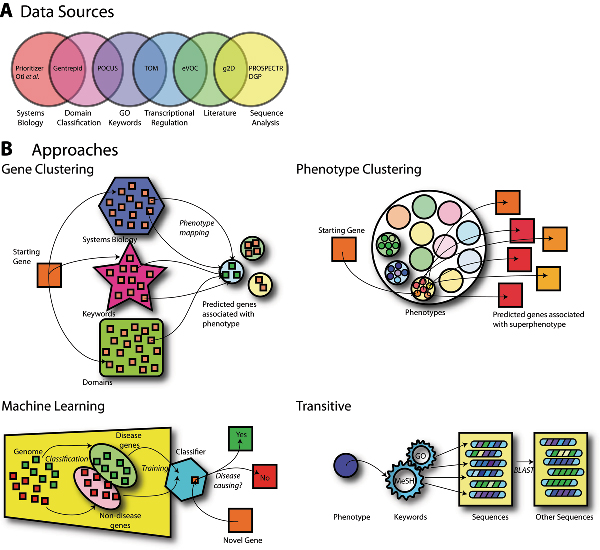
**Data sources and approaches used in automated candidate gene prediction methods**. **(A): **Most systems draw on at least two types of data. *SUSPECTS *[21] (not shown) uses keywords from InterPro [22] and GO [23], co-expression data, and also incorporates the *PROSPECTR *module [12] (shown on right). **(B)**: *Upper left *Gene clustering approaches associate a gene cluster with a phenotype via a group member. For example, Systems Biology approaches [4,5,24] group genes whose protein products interact; and link them to a phenotype using a group-member gene associated with the phenotype. Systems Biology methods assume oligogenic diseases are associated with disruption in proteins that participate in a common complex or pathway [25]. Other gene clustering systems look for enrichment of keywords or domains associated with particular phenotypes and suggest candidate genes with similar properties. These systems are based on the principle that candidate genes have similar functions to disease genes already determined [26-28]. *Upper right *Phenotype clustering approaches such as that of Freudenberg & Propping [29] group related phenotypes into superphenotypes. *Lower left *Most of the Machine Learning approaches do not use phenotype information and are based on the concept that the genome consists of a bipartite distribution of genes: those which cause diseases, and those that do not. By analysing these two gene sets with respect to discriminating variables, a profile for "non-disease genes" and "disease genes" is produced which enables training of a classifier. A novel gene submitted to the classifier is flagged as either "disease-causing" or "non-disease causing". Systems include *eVOC *[30], *PROSPECTR *[12], *SUSPECTS *[21] and *DGP *[31]. Finally *G2D*, *lower right*, is a transitive method that maps phenotypes to genes [32] by interfacing literature- and keyword-based ontologies.

Candidate gene prediction systems vary in their approach and the data sources they draw on in generating predictions. These are summarised in Figure 1 and Table 1. Comparing the performance of these systems can be difficult because of the use of custom benchmark test sets by individual groups. Typically, benchmarking data is derived from genotype-phenotype information from the Online Mendelian Inheritance in Man (OMIM) database [2], but groups have used varying subsets of diseases. Several groups have tried to use standard benchmark sets [3-5], but these efforts have been limited. In addition, it is difficult to predict whether benchmarks which predominately contain data on well characterised diseases with Mendelian transmission patterns (i.e. dominant, recessive, X-linked) resulting from mutations in single genes [6] will be effective in predicting genes involved in less well characterised diseases, or in complex diseases.

**Table 1:** Automated Candidate Gene Prediction Systems

**Semi-Automated Systems**
*GeneSeeker *is a semi-automated web-server tool which selects positional candidates based on expression and phenotypic data from human and mouse. Queries must be formulated by the end-user using Boolean expressions [13,33]. ♠ ◇
**Systems Biology Techniques**
*Prioritizer *uses pathway and interaction data from KEGG [17,34], Reactome [35], and HPRD [36]. Interactions are also predicted using a Bayesian technique based on GO keywords [23] and other databases [5].
In *Gentrepid *Common Pathway Scanning (CPS), pathways are associated with phenotypes using either known disease genes, or by searching for enrichment of pathways across multiple disease intervals associated with the phenotype [4]. ♠◇
*Oti et al *use protein-protein interaction data from HPRD [36], Y2H [37,38], and PCP [39,40] giving coverage of 10 894 human genes [24].
**Genotype-Phenotype Mapping Methods**
*G2D *[32] uses biomedical literature to associate pathological conditions with GO terms [23]. Candidate genes are identified by homology to GO-annotated disease-associated genes. ♠◇
*Gentrepid *Common Module Profiling (CMP) searches for enrichment of particular domains in gene clusters associated with a particular phenotype. Domains are extracted either from known disease genes or by comparison of multiple disease intervals [4]. ♠◇
*POCUS *searches for over-representation of functional annotation among multiple loci associated with the same disease. Functional annotation is based on keywords from InterPro domains [22] and GO [23]. No *a priori *knowledge of the phenotype is required [3]. ♠
**Techniques based on a bipartite distribution of "disease" and "non-disease" genes**
The *eVOC *system uses text mining of biomedical literature to associate a phenotype with anatomy terms and links these with human expression data to produce a ranked list of disease genes. The classifier is a machine-learning technique, based on a bipartite training set of 17 known "disease genes" and 306 "non-disease genes" [30]. ♠
*DGP (Disease Gene Prediction) *is a web tool which selects genes based on protein sequence properties. The properties analysed by DGP include protein length, degree of sequence conservation, the extent of phylogenetic relationship and paralogy patterns [31,41]. ♠
*PROSPECTR *(PRiOrization by Sequence and Phylogenetic Extent of CandidaTe Regions) uses an alternating decision tree to discriminate "disease genes" from "non-disease genes" using a classifier based on sequence features such as gene length, protein length, and similarity of homologs in other species [12]. ♠
**Hybrid techniques**
*SUSPECTS *combines a genotype-phenotype mapping method based on disease-gene-associated keywords from InterPro and GO, and expression libraries, with the *PROSPECTR *Boolean classifier. Disease genes are prioritized [21]. ♠ ◇

♠ Assessed here, ◇ Webserver.

A recent effort by Tiffin and colleagues [7] to identify candidate disease genes for the complex disease type II diabetes (T2D) and the related obesity trait predicted 12 genes in previously implicated chromosomal regions. The study also allowed a limited comparison of seven candidate gene prediction systems. Since that time two genome-wide association studies (GWAs) on T2D undertaken by the Wellcome Trust Case Control Consortium (WTCCC) and the Genetics Replication and Meta-analysis Consortium (DIAGRAM) have been published [8,9]. GWAs are a powerful tool for identifying genetic variants linked to complex diseases because they are more sensitive than linkage studies to small to moderate effect size contributions from polygenic and oligogenic diseases. The data from these GWAs allow the assessment of the predictions made by Tiffin *et al*., as well as evaluation of the effectiveness of predictions made by the individual automated candidate gene prediction systems used in their study and our system, *Gentrepid *[4]. We assessed the candidate gene predictions systems' ability to select robustly supported genes from the GWAs and used them to filter noisy data from statistically less well supported genes to select favoured candidates.

## Results

### Predictions

All methods were given the starting set of 9556 genes mapped to chromosomal intervals implicated in T2D as assessed by Tiffin *et al*., except for *POCUS *which was run against a search space of 562 genes. The *POCUS *method was confined to the smaller search space because "poorly defined susceptibility regions or regions with questionable association with the disease are obscured by background noise" [7]. The number of candidate gene predictions made by the eight methods varied from two to 3093. *POCUS *generated the smallest number of candidates but neither of the two predictions matched genes in either the highly significant (HS) or medium-to-highly significant (MHWD) data sets. Other candidate gene prediction methods made considerably more predictions. The largest numbers of predictions were made by *G2D *(3,093 candidate gene predictions) and *eVOC *(2,496 predictions). These comprise almost one third and one quarter of the search space respectively. Thus neither of these methods prune the search space particularly well. Excluding *POCUS*, the least number of predictions was made by *Gentrepid *comprising 502 genes in known-disease-gene mode.

### Accuracy of predictions

**Figure 2:**
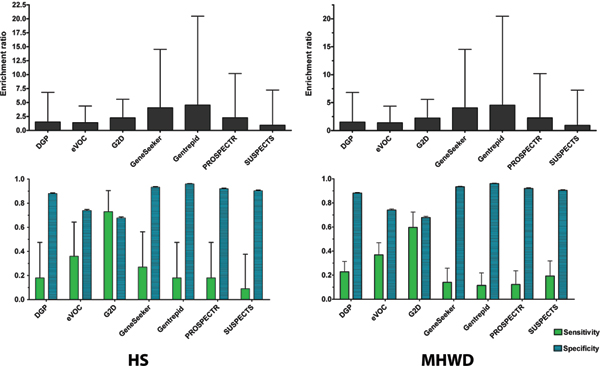
**Comparison of methods against the HS (left) and MHWD (right) T2D gene data sets**. Top: Relative Enrichment Ratios. Bottom: Comparisons based on Sensitivity and Specificity.

To assess the accuracy of the predictions, all eight systems were compared with genes found in previously-implicated intervals strongly linked to T2D by the GWAs. Figure 2 shows the comparative performance of seven of these methods in selecting the 11 genes in the HS GWA data set. Several metrics were calculated to assess accuracy. No metrics were calculated for *POCUS *as neither of its two predictions matched genes in either the HS or MHWD data sets.

The Enrichment Ratio is a general measure of the system's ability to accurately prune the search space. Enrichment Ratios ranged from 1 to 5 for the seven remaining prediction systems. The highest Enrichments Ratios were obtained by *Gentrepid *and *GeneSeeker*. These results were robust when the upper and lower (not shown) 95% confidence interval limits were taken into account. The lowest Enrichment Ratios were associated with the Machine Learning methods. This is not surprising, as the classifiers are trained to distinguish "disease genes" from "non-disease genes" and are ignorant of any concept of phenotype. The Specificity of a system measures its ability to reject genes not associated with the phenotype. Specificity scores among all seven methods ranged from 0.68 to 0.99, with a median of 0.92. As a group, the Machine Learning methods were poorer at rejection. *G2D *also performed poorly on this metric, but this result is slightly misleading because it does not take into account *G2D*'s prioritization method which will be discussed later.

The Sensitivity is a measure of a system's ability to find the disease genes in the search space. A caveat here is not all of the GWA predictions are currently confirmed. *G2D *is by far the standout performer in Sensitivity, with *eVOC *ranked second. However, as can be seen from the other metrics, this result is obtained at the expense of Specificity for both systems. *Gentrepid's *Sensitivity is on par with most of the Machine Learning methods but with higher Specificity. The high Specificity reflects the high quality of the data in the underlying databases. The lower Sensitivity is due to incompleteness of these databases with respect to all human genes.

Figure 2 shows the comparative performance of methods when assessed against the 61 T2D associated genes with moderate to strong SNP signals (MHWD) in the Tiffin chromosomal intervals. The MHWD data set is not as statistically well supported as the HS set, and would be expected to contain some genes associated with T2D and others that are false positives. Perhaps the most interesting metric to look at here is the Sensitivity which should fall compared to the values for the HS set because of the lower signal to noise ratio in the MHWD set. All the systems except one, *SUSPECTS*, passed this negative test. More importantly, application of the systems to this noisy genetic data allows selection of a subset of candidates on the basis of molecular data (see below).

The results shown for *Gentrepid *in Figure 2 are for the known-disease-gene mode. In *ab initio *mode, *Gentrepid*'s CPS method identified 506 pathways containing a total of 1980 candidate gene predictions. This resulted in Enrichment Ratios of 3.3 and 2.1 when the HS and MHWD full gene sets were considered (Table 2).

**Table 2:** Gentrepid *ab initio *results

Predictions	Reference list	ER	L95%	U95%	S	L95%	U95%
CPS rank 8+ pathways	HS	3.3	1.1	9.4	0.45	0.21	0.72
CPS rank 8+ pathways	HS – annotated	7.2	2.1	25	1.00	0.57	1.00
CPS rank 8+ pathways	MHWD	2.1	1.3	3.6	0.30	0.20	0.42
CPS rank 8+ pathways	MHWD – annotated	6.8	3.6	13	0.95	0.75	0.99
							
CPS interactions top 50%	HS	4.1	1.2	15	0.27	0.10	0.57
CPS interactions top 50%	HS – annotated	9.0	2.2	37	0.60	0.23	0.88
CPS interactions top 50%	MHWD	1.7	0.79	3.8	0.11	0.06	0.22
CPS interactions top 50%	MHWD – annotated	8.1	3.2	20	0.54	0.29	0.77
							
CMP top 10%	HS	2.2	0.3	17	0.1	0.02	0.38
CMP top 10%	MHWD	2.0	0.8	4.8	0.1	0.08	0.18

Abbreviations in Table: ER – Enrichment Ratio, L95% – Lower 95% confidence limit, U95% – Upper 95% confidence limit, S – Sensitivity

In *ab initio *mode, the CMP method generated 527 predictions by limiting the selection to the top 10% most probable genes. This resulted in correct prediction of one gene from the HS set and five from the MHWD set, yielding a Enrichment Ratio of 2.2 when applied to the HS and 2.0 for the MHWD gene data sets. 

It is also interesting to note the effect of lack of annotation on these results. Only five of 11 genes in the HS dataset, and 19 of 61 genes in the MHWD set contained KEGG or BioCarta pathway annotations.When we included only genes containing pathway information from the gene datasets (designated 'annotated' in Table 2) we observed Enrichment Ratios of 7.2 against the HS and 6.8 against the MHWD pathway-annotated sets. Sensitivities also improved by a factor of 2 for the HS dataset. By extrapolation, if all genes were pathway annotated, we could expect approximately two- to three-fold improvement in Enrichment and Sensitivity scores.

### Prioritization

Although the metrics discussed provide useful measures of a candidate gene prediction system's performance, another criterion of importance to geneticists is the system's ability to prioritize predictions. Although several methods claim the ability to prioritize (Table 1), only *G2D *provided prioritized predictions in Tiffin *et al*. [7]. Hence only *G2D *and *Gentrepid *will be discussed here. Because *Gentrepid *only made 502 predictions *in toto*, we took the top 502 predictions made by *G2D *and recalculated the Enrichment Ratio, Sensitivity and Specificity for this restricted set of favoured predictions. ER and Specificity significantly improved to 3.1 and 0.95 such that *G2D *surpassed *Gentrepid*'s gross ER and almost equalled *Gentrepid *in Specificity. The improvement in these two metrics came at the expense of Sensitivity which was reduced to 0.16, but the *G2D *system still managed to maintain its lead on this metric.

In *G2D*'s prioritization system, a GO-metric is calculated for each gene in the search space based on how well its GO profile fits the GO profile of the disease genes inferred from MeSH terms. An R-score is calculated for each gene by normalizing against the number of genes in the genome with better GO-metrics for the phenotype. Genes with R-scores closer to zero are better fits to the phenotype.

*Gentrepid *CPS ranks genes by the number of loci in the search space involved in a particular pathway. In *ab initio *mode, of the 53 intervals searched, the top pathway, focal adhesion, was represented in 35 of these. All five of the HS dataset genes were represented by pathways found in at least eight intervals. Pathways implicated in at least eight intervals constituted the top 40% of the 506 pathways containing 1749 candidates. In these pathways, *Gentrepid *identified all five pathway annotated genes from the HS dataset, and 18 of the 19 pathway annotated genes from the MHWD gene data set. Other figures for CMP are given in Table 2.

**Figure 3:**
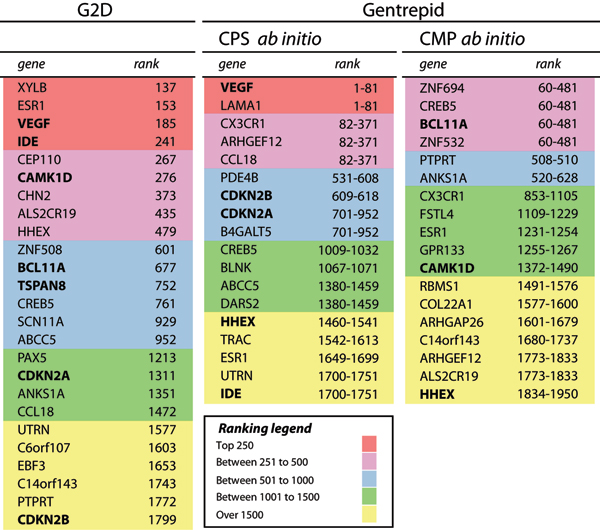
**MHWD dataset filtered against prioritized automatic candidate gene predictions**. Genes in bold are robustly supported genes from the GWA studies (HS set).

CMP *ab initio *looks for protein domains enriched in the search space compared to the genome by taking a census of domains in the search space and the genome. Two expectation values are calculated to estimate the frequency of occurrence of genes with domains of interest based on a random combination of these domains *e*_*a *_and the rarest domain *e*_*b *_[4]. Figure 3 shows the data ranked on a *χ*^2 ^test based on e_a _was most effective in prioritizing the HS data. This reflects our experience of phenotypes with genotypes encoded by multi-domain proteins, as would be expected for diseases associated with signaling. For metabolic diseases associated with single-domain proteins, *e*_*b *_may be a better measure.

Although the *G2D *prioritization system appears more sensitive than the coarse-grained prioritization of *Gentrepid*, the performance of both systems was roughly equivalent against the HS set. Both systems were moderately successful in prioritizing the HS data. For example, of the seven genes in the HS dataset predicted by *G2D*, four were ranked in the top 15% by *G2D*'s prioritization method (bold in Figure 3). Significant work needs to be done to improve the prioritization schemes of both *G2D *and *Gentrepid*. 

Finally, we used the candidate gene predictions systems to filter the less statistically-well-supported MHWD data set (MHWD – HS): effectively adding more power to the GWA study. Prioritized predictions are the unbolded genes in Figure 3. Additional unprioritized predictions made for the MHWD dataset using the other candidate predictions systems are given as supplementary data in Additional file 1.

## Discussion

Candidate disease gene prediction is a rapidly moving area of bioinformatics research with the potential to deliver great benefits to human health. By assisting geneticists to use existing biological information to investigate disease loci obtained by linkage analysis and association studies, disease genes can be identified more rapidly. The need for good applications in the area of candidate gene prediction is becoming increasing important as the proliferation of SNP-based association studies produces valuable genetic information in need of analysis.

The biggest problem facing candidate gene prediction today is the accuracy and completeness of the underlying databases. Failure to make a prediction is mostly due to incomplete data coverage. For example, 65% of human proteins have GO terms but only 25% of these are manually annotated. Systems drawing on GO terms like *G2D *are potentially able to make predictions for 65% of genes but only around one third of these are likely to be accurate. Systems Biology methods like *Gentrepid *CPS are reliant on pathway and protein-protein interaction data. One of the databases CPS draws on is OPHID [10], one of the most complete protein-protein interaction datasets, containing over 48 000 interactions. However these 48,000 interactions are estimated to be only 13% of the complete human interactome [11]. Completeness of the underlying data clearly impacts the Sensitivity of the *Gentrepid *CPS method. As time goes on this constraint will ease as these databases are further populated. In the meantime, we have shown that the use of independent biological data to make complementary candidate gene predictions is one way to ameliorate the problem of incomplete data coverage (see Figure 3) [4].

In addition to the predictions made by the individual candidate gene prediction systems in Tiffin *et al*., a set of nine "winners" were chosen using a consensus approach [7]. These nine candidate genes were independently predicted by six of the seven prediction systems studied. A larger consensus set, chosen by five of the seven methods, contained 94 genes [7]. None of the genes in either of these consensus lists matched any of the genes in the HS and MHWD gene sets. Even if we compile a third tier of consensus genes from any four of the seven methods (269 genes) only one gene (VEGF) fell within the HS data set and only three genes (CHN2, B4GALT5, VEGF) matched the MHWD data set. Clearly the consensus approach is not working and it is easy to see why when the underlying databases are considered (Figure 1A). Candidate gene prediction systems that use an independent data set, not drawn upon by most of the other methods, will be penalized. Possibly the only benefit of a consensus approach is to give the user a false sense of accuracy when confronted with noisy data.

Clearly much work still remains to improve the sensitivity and specificity of candidate gene prediction methods but some general conclusions are possible. Machine Learning methods were not as effective as other methods. Most of the Machine Learning approaches do not use phenotype information and are based on the concept that the genome consists of a bipartite distribution of genes: those which cause diseases, and those that do not. The evidence supporting this assumption is limited [12]. We believe the concept that there is a difference between "disease genes" and "nondisease genes" is intrinsically flawed and no such Boolean classification exists. We hypothesize that the ability of these methods to predict disease genes in test sets is based on selection effects in the data: possibly rare, highly penetrant monogenic diseases, such as those involved in metabolic syndromes, are over-represented among known disease genes because they have been easier targets to identify. Although these systems were not as effective as the other candidate gene prediction systems, their performance was not greatly different. However, we believe that unlike systems which attempt to map genotype to phenotype, Machine Learning systems based on the disease gene/non-disease gene concept will not improve as more biological data becomes available.

## Conclusion

Candidate gene prediction systems have typically been benchmarked on well characterized oligogenic phenotypes. GeneSeeker [13] produced a 10-fold enrichment using a data set consisting of eight diseases. *Gentrepid*'s combined methods [4] produced an Enrichment Ratio of 13 when 29 diseases with a total of 170 known disease genes were used. For 29 diseases with 163 genes, POCUS [3] reported Enrichment Ratios between 12 to 42-fold, depending on the size of the intervals in the search space. The PRIORITIZER [5] method yielded a 2.8-fold enrichment using a data set consisting of 96 heritable disorders. In summary, Enrichment Ratios of 3 to 13 have been reported in benchmarks, but a substantial part of the data used for these studies has been limited to oligogenic phenotypes, where several different genes may cause the disease, but a single mutation in each case or family has a large effect.

Some doubts have been raised about the ability of systems to predict candidates for complex polygenic diseases such as T2D where multiple genes interact to create a permissive gene pool for disease genesis. The candidate gene prediction systems did prune the genome in favour of moderately to highly significant SNPs identified by the GWAs under semi-blind testing on a complex polygenic disease. Enrichment Ratios calculated in this study suggest that most of the oligogenic benchmarks have been reasonably good predictors of system performance.

## Methods

### Benchmark datasets

Eight candidate gene prediction systems were assessed on their ability to predict genes involved in T2D by comparison against genes implicated by recent GWAs. Two data sets of T2D-implicated genes were used as the benchmark: a **H**ighly **S**ignificant gene set (HS) of 21 genes and a **M**oderate to **H**ighly significant gene set derived from the **W**TCCC and **D**IAGRAM studies (MHWD) of 172 genes [8,9]. The HS gene set contained 11 genes which mapped to the chromosomal regions investigated by Tiffin *et al. *(hereafter Tiffin intervals) [7]. Genes associated with 706 moderately significant SNPs with a frequentist additive p-value of <0.001, good clustering and intact NCBI build 36 reference ids were taken from WTCCC T2D data [8]. SNPs positioned between the 5' UTR and 3' UTR of a known gene structure, 1000 bases upstream of a 5' UTR or 1000 bases downstream of 3' UTR of a known gene were considered to implicate the gene in T2D disease susceptibility. This moderately significant list was combined with the genes from the HS data set to generate the MHWD dataset, yielding 172 genes genome wide of which 61 genes mapped to the Tiffin intervals [7].

### Data sources for predictions

The search space available to all eight automated candidate gene prediction systems consisted of 9556 genes in 53 chromosomal loci assessed by Tiffin *et al*. to be involved in T2D by various linkage and association studies. We matched 96.5% of all Ensembl gene entries [14] provided to NCBI Entrez ids. All remaining genes were unable to be matched due either to the Ensembl entry having an unknown gene symbol label or because the entry was ambiguous or associated with a redundant gene symbol name entry. Ensembl entries and NCBI id matching was carried out at four levels, in order: approved symbol name, previous symbol names, Uniprot/SwissProt Accession and RefSeq Ids. Data conversion keys for matching between databases were acquired from BioMart [15].

Predictions made by seven candidate gene prediction methods were also obtained from Tiffin *et al*. [7]. Nine disease-implicated genes were available to the systems as seeds (PPARG, GYS1, IRS1, INS, KCNJ11, ABCC8, SLC2A1, PPARGC1, CAPN10). Two of the genes – PPARG and KCNJ11, are implicated by the highly significant SNPs detected by GWAs but are not in the Tiffin intervals and are thus not included in the search space or benchmark set.

### Candidate gene predictions

The candidate gene predictions for seven of the systems are detailed elsewhere [7]. Briefly, *GeneSeeker *selected genes from the search space using a Boolean expression based on 14 keywords selected by an expert user [7]. *PROSPECTR*, *DGP *and *eVOC *are Boolean classifiers which require only the search space as input. *G2D *and *Gentrepid *in *ab initio *mode, also only require the search space. *POCUS *potentially only needs the search space as input, but this was restricted to the seven best supported intervals of the 53 available, as judged by the *POCUS *team. *SUSPECTS *and *Gentrepid*, in known-disease-gene mode, used the nine known disease genes associated with the phenotype as seeds. *SUSPECTS *additionally draws on predictions from the *PROSPECTR *Boolean classifier.

*Gentrepid *predictions are discussed in detail here for the first time. *Gentrepid *implements two different modules to derive predictions: CPS – a systems biology method; and CMP – a method that associates phenotypes with particular domains. CPS and CMP can be used in two input modes: using known disease genes as a seed or using only the search space (*ab initio *mode).

In known-disease-gene input mode, CPS searches all pathway and interaction data in BioCarta [16], KEGG [17] and I2D (formerly OPHID) [10] to extract all genes associated with the disease gene, and then filters this list against implicated loci. Genes are ranked based on the total number of genes implicated in the pathway. For example, if two known disease gene seeds and three genes in the loci being investigated are found in the same pathway, the pathway is given a rank of five against the phenotype. CMP parses the protein sequences of the known disease genes associated with the phenotype into domains using the Pfam library of Hidden Markov Models (HMMs) [18] and then retrieves any other genes with related domain content from the genome. A score between 0 and 1 is generated reflecting the candidate gene's similarity to a known disease gene [4]. The same nine disease genes and 53 chromosomal cytogenetic bands were used by *Gentrepid *as per Tiffin *et al*..

In *ab initio *mode, *Gentrepid *can make predictions in the absence of known disease genes if two or more loci are provided as input. *Gentrepid*'s CPS *ab initio *method is based on the premise that pathways whose genes are more prevalent within disease-implicated loci (chromosomal regions) compared to the entire genome have a higher probability of involvement in the pathoetiology of the disease phenotype of interest. Analogous to the known disease gene mode, pathways are ranked by the number of loci involved. The CMP *ab initio *method searches for enrichment of domains in the loci with respect to the genome and ranks genes based on the statistical significance of the domain enrichment (equations 2 and 4 in [4] where *mn *is replaced by Σ – the total number of genes in the intervals examined).

For each input mode, a final list of predictions is made by consolidating all predictions from both the CMP and CPS modules.

### Metrics for comparisons

Systems were compared using three metrics: Enrichment Ratio, Sensitivity and Specificity. The Enrichment Ratio calculations were calculated as below [4]:

(1) $$EnrichmentRatio=\frac{TP/(TP+FP)}{\sum genes_{implicated}/\sum genes_{all}}$$

The denominator was obtained by dividing the number of T2D implicated genes by the total number of genes within all surveyed chromosomal regions.

Sensitivity and Specificity were calculated as below:

(2) $$Sensitivity=\frac{TP}{\left(TP+FN\right)}$$

(3) $$Specificity=\frac{TN}{\left(TN+FP\right)}$$

TP is the number of true positives, FP is the number of false positives, TN is the number of true negatives and FN is the number of false negatives. Sensitivity is the proportion of true positives among all disease genes in the chromosomal regions. Specificity is the proportion of true negatives among genes not associated with the disease in chromosomal regions. Confidence intervals were estimated using the method of Newcombe [19] implemented using the CIcalculator software [20].

## List of abbreviations used

T2D: Type II diabetes; WTCCC: Welcome Trust Case Control Consortium; DIAGRAM: Genetics Replication and Meta-analysis Consortium; SNP: Single nucleotide polymorphism; GWA: Genome wide association studies; HPRD: Human Protein Reference Database; BIND: Biomolecular Interaction Network Database; DGP: Disease Gene Prediction; PROSPECTR: PRiOrization by Sequence and Phylogenetic Extent of CandidaTe Regions; OMIM: Online Mendelian Inheritance in Man; OPHID: Online Predicted Human Interaction Database; KEGG: Kyoto Encyclopedia of Genes and Genomes; GO: Gene Ontology; MeSH: Medical Subject Headings; HS: Highly Significant gene set; MHWD: Moderate to Highly gene set derived from WTCCC and DIAGRAM studies; ER: Enrichment Ratio; S: Sensitivity; TP: true positives; FP: false positives; TN: true negatives; FN: false negatives.

## Competing interests

The authors declare that they have no competing interests.

## Authors' contributions

MW design, concept and oversight of study, and manuscript author. ET manuscript author, and data generation for study. JL implementation, construction and maintenance of database. SB additional data generation, figures and manuscript preparation. DF Genetics consultant. All authors read and approved the final manuscript.

## Supplementary Material

Additional file 1 **Additional unprioritized MHWD matches**. Additional unprioritized MHWD matches data. Click here for file
